# Autoantigen Microarray for High-throughput Autoantibody Profiling in Systemic Lupus Erythematosus

**DOI:** 10.1016/j.gpb.2015.09.001

**Published:** 2015-09-28

**Authors:** Honglin Zhu, Hui Luo, Mei Yan, Xiaoxia Zuo, Quan-Zhen Li

**Affiliations:** 1 Department of Rheumatology and Immunology, Xiangya Hospital, Central South University, Changsha 410008, China; 2 Department of Immunology and Internal Medicine, University of Texas Southwestern Medical Center, Dallas, TX 75235, USA

**Keywords:** Systemic lupus erythematosus (SLE), Autoantibody profiling, Proteomic microarray, Biomarker, High-throughput assay

## Abstract

**Systemic lupus erythematosus (SLE)** is a complex autoimmune disease characterized by the production of autoantibodies to a broad range of self-antigens. Profiling the autoantibody repertoire using array-based technology has emerged as a powerful tool for the identification of **biomarkers** in SLE and other autoimmune diseases. **Proteomic microarray** has the capacity to hold large number of self-antigens on a solid surface and serve as a high-throughput screening method for the determination of autoantibody specificities. The autoantigen arrays carrying a wide variety of self-antigens, such as cell nuclear components (nucleic acids and associated proteins), cytoplasmic proteins, phospholipid proteins, cell matrix proteins, mucosal/secreted proteins, glomeruli, and other tissue-specific proteins, have been used for screening of autoantibody specificities associated with different manifestations of SLE. Arrays containing synthetic peptides and molecular modified proteins are also being utilized for identification of autoantibodies targeting to special antigenic epitopes. Different isotypes of autoantibodies, including IgG, IgM, IgA, and IgE, as well as other Ig subtypes, can be detected simultaneously with multi-color labeled secondary antibodies. Serum and plasma are the most common biologic materials for autoantibody detection, but other body fluids such as cerebrospinal fluid, synovial fluid, and saliva can also be a source of autoantibody detection. Proteomic microarray as a multiplexed high-throughput screening platform is playing an increasingly-important role in autoantibody diagnostics. In this article, we highlight the use of autoantigen microarrays for autoantibody exploration in SLE.

## Introduction

Systemic lupus erythematosus (SLE) is a prototype of chronic autoimmune connective tissue disease with an insidious onset that can affect almost every system and organ in the human body, especially the musculoskeletal, renal, cardiovascular, mucocutaneous, and central nervous systems [1–3]. SLE has diverse manifestations accompanied by a large number of autoantibodies. So far, more than 180 autoantibody specificities have been found in the blood of SLE patients, although different patients may exhibit different autoantibody profiles [4–6]. Circulating autoantibodies can be detected years prior to the clinical onset of SLE and in some patients the number of distinct autoantibodies was found to increase over time [7,8]. It is conceivable that some of the autoantibodies play pathogenic roles and are associated, at least in part, with the wide spectrum of clinical manifestations in SLE [9,10]. Currently, the clinical diagnosis of SLE relies on the presence of at least 4 out of the 11 criteria suggested by the American College of Rheumatology (ACR) [1]. The presence of anti-nuclear antibodies (ANA) in patient’s serum is the most important laboratory criteria for SLE diagnosis. ANA represents a cluster of autoantibodies targeting to various components of the cell nucleus and is positive in over 90% of SLE patients [11]. However, about 20% of the general population can show ANA positivity in their sera, and among them approximately 2.5% of unaffected individuals may have very high ANA levels [11,12]. ANA is also present in other autoimmune diseases, such as Sjogren’s syndrome (SS), scleroderma, rheumatoid arthritis, and mixed connective tissue disease (MCTD). Thus, although ANA has been used as a serological marker for diagnosis of SLE for many years, its value has been sometimes discounted due to its poor specificity [12].

Autoantigen array as a high-throughput autoantibody screening platform has the potential to distinguish autoantibody specificities against a wide spectrum of autoantigens and is therefore valuable for the evaluation of the correlation between autoantibodies and clinical manifestations [13–16]. Previous studies have shown that autoantibodies can exist in sera of SLE patients many years prior to the onset of clinical disease and the number of autoantibodies correlated with disease severity [7,8]. Hence, profiling autoantibodies using high-throughput autoantigen arrays may have important implications for early diagnosis and prognosis of SLE.

## Autoantigen microarray: principle and methodology

The principle of proteome microarray was firstly described by MacBeath and Schreiber who developed a miniaturized assay, which had the capacity to accommodate thousands of proteins [17]. They used a high-precision contact-printing robot to deliver nanoliter volumes of protein onto chemically-derivatized glass slides. The proteins were covalently attached to the substrate coated on the slide surface, which retained their activity to interact with other proteins, or small molecules, in solution. As a proof-of-principle test, they demonstrated three applications for protein microarrays: to screen for interactions between proteins, to identify the substrates of protein kinases, and to identify the small molecules as targets of the proteins [17]. The important application of proteomic microarray is for high-throughput quantitative detection of the interactions between specific antigens and antibodies in complex solutions. Haab et al. analyzed the specificity, sensitivity, and accuracy of protein array on 115 antibody/antigen pairs. Over 50% of the arrayed antigens were specifically detected by their corresponding antibodies with the sensitivity at or below the concentration of 0.34 μg/ml. Moreover, some even allowed detection of the cognate antibodies at absolute concentrations below 1 ng/ml, which is sensitive enough for measurement of many clinically-important proteins in patients’ blood samples [13].

**Figure 1:**
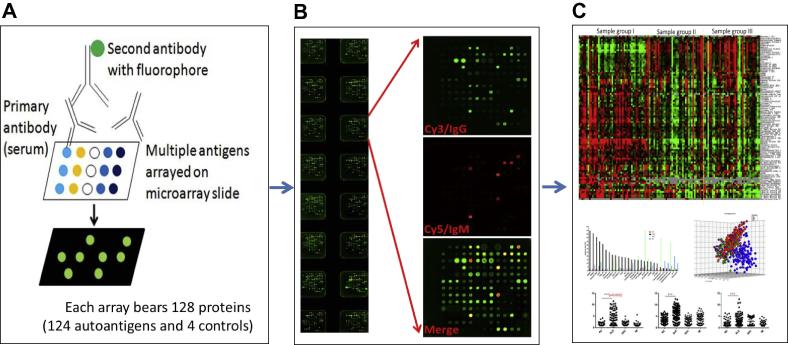
**Autoantigen microarray for high-throughput autoantibody screening** **A.** Mechanism of autoantigen microarray. Autoantigens are immobilized onto nitrocellulose-coated slides, after hybridization with samples, the autoantibodies bound with autoantigens are detected with fluorescent dye-labeled secondary antibodies. **B.** Image of multiplex autoantigen arrays for detection of human IgG and IgM autoantibodies. Each slide has 16 identical arrays of 128 antigens. Arrays are hybridized with human sera, detected by Cy3-labeled anti-human IgG and Cy5-labeled anti-human IgM antibodies, and scanned with Axon 4000B scanner. **C.** Autoantigen microarray data analysis. Heatmap (top panel) is generated by Cluster and TreeView software using the signal intensity of the autoantibodies to all samples. The graphs in the lower panel show the statistical analysis using Prism 6 software.

Autoantigen array is a specified proteome microarray for large-scale detection of autoantibodies on the basis of antigen–antibody reaction as shown in Figure 1. Autoantigen can be any of an organism’s own antigens (self-antigens), *e.g.*, nuclear antigens, cytoplasmic antigens, cell membrane antigens, phospholipid-associated antigens, blood cells, endothelial cells, glomerular basement membrane, mitochondria, muscle, parietal cells, thyroglobulin, nervous system antigens, plasma proteins, matrix proteins, and miscellaneous antigens, which may evoke production of autoantibodies. The common autoantigens identified in SLE are listed in Table 1. Among them, the nuclear antigens are the most popular autoantigens targeted by autoantibodies in SLE. The methodology underlying the autoantigen microarray has been reviewed previously [16,18] and is briefly described as follows. The autoantigen arrays are produced by immobilizing hundreds or even thousands of diverse autoantigens on the coated surface of glass slides. The autoantigens can be nucleotide (DNA or RNA) or purified proteins from tissues, *in vitro*-expressed recombinant proteins, or synthetic peptides and the glass slides can be coated with nitrocellulose membrane (NC), hydrogel, or polymers, which hold the proteins in their native conformation. After blocking, the arrays are hybridized with diluted biological samples (serum, body fluids, or cell culture supernatant), and finally the autoantibodies bound to their corresponding antigens on the array are detected with the fluorophore-conjugated second antibodies against different isotypes of autoantibodies (IgG/IgM/IgA/IgE). Shown in Figure 1 is the multiplex autoantigen microarray chip, which can process 16 samples on one chip in one run and detect 125 autoantibodies for both IgG and IgM isotypes.

There are some obvious advantages of the autoantigen array over the conventional ANA detection methods such as indirect immunofluorescence (IF) and ELISA [19]. Use of the aforementioned conventional methods to analyze multiple antibodies in multiple samples may incur substantial cost, time, manpower, and even the serum samples. In contrast, autoantigen arrays can be easily performed as high-throughput assays, using smaller volume of serum (1–2 μl) and at much lower cost. Most significantly, autoantigen array has the capacity to detect the specificities of hundreds even thousands of autoantibodies in a quantitative manner. It has been demonstrated that data generated by autoantigen arrays were correlated very well with the data generated by ELISA, however with much higher sensitivity [20–22].

## Autoantigen arrays for multiplex characterization of autoantibodies

Autoantigen microarray for large-scale detection of autoantibody responses was first reported by Robinson and colleagues [20]. They fabricated 1152-feature arrays containing 196 putative autoantigens, which have been reported to be targeted by autoantibodies in different autoimmune disorders. These included 36 recombinant or purified proteins and 154 overlapping and immunodominant peptides of the autoantigens. The autoantigen arrays, which were incubated with a mixture of sera derived from patients with SLE, polymyositis (PM), or primary biliary cirrhosis (PBC), specifically identified autoantibodies recognizing mammalian double-stranded DNA (dsDNA), synthetic single-stranded DNA (ssDNA), histone H2A, U1 small nuclear ribonucleoprotein (U1-snRNP), Smith antigen (Sm), Sm/RNP complexes, Ro52, Jo-1, and pyruvate dehydrogenase (PDH). Moreover, the autoantigen arrays showed a consistently 4–8-fold higher sensitivity than ELISA for detecting antigen specific autoantibodies [20].

Using the same autoantigen arrays, this group also detected isotype-specific mouse autoantibodies. They measured IgG1 and IgG2a antibody isotype in a murine model of autoimmunity using isotype-specific secondary antibodies labeled with Cy3 and Cy5. As a result, they demonstrated that the autoantigen array can quantitatively monitor changes in isotype mAb concentration [21]. Thus, autoantigen microarray technology has been shown to be a sensitive and specific assay for quantitative measurement of antibody subclasses in biological samples, such as serum, cerebrospinal fluid (CSF), peritoneal fluid, and synovial fluid [20,21].

## Autoantigen arrays for profiling autoantibodies in SLE and incomplete LE

To provide a comprehensive understanding of the autoantibody repertoires during the development of SLE, Li and colleagues developed protein microarrays comprising a collection of autoantigens related to various autoimmune disorders [22,23]. Using these antigen microarrays, they identified autoantibody clusters associated with overall disease activity and lupus nephritis (LN) [22]. They further analyzed the autoantibody profiles in a subset of patients with incomplete LE (ILE), defined as having at least one but less than four of the SLE diagnostic criteria and in first-degree relatives (FDR) with SLE. By comparing the serum levels of IgG and IgM autoantibody isotypes in the subgroups of healthy controls (HCs), ILE, and SLE, they found that patients in SLE group exhibited increased level of IgG autoantibodies compared with ILE, whereas alterations in IgM autoantibodies showed the opposite trends: high in ILE but low in SLE, implying that there might be a class switch from IgG to IgM during the transition from ILE to SLE [23]. Furthermore, in combination with transcriptional profiling, they found the association between the peripheral blood interferon (IFN) signature and serum autoantibodies in patients with SLE and ILE. High expression of IFN signature genes were significantly correlated with high levels of IgG autoantibodies. Therefore, IFN may play a pathogenic role in driving IgM to IgG class-switching in SLE [24].

The same group also examined the autoantibody profiles in ANA-positive healthy population using an autoantigen array carrying over 100 antigens. They found that healthy population with high ANA showed significant elevation of autoantibodies against antigens in skin, kidney, thyroid, or joints. The profiling of autoantibodies in high ANA population, in combination with other clinical features, may help to identify individuals who are at higher risk of developing SLE [25,26].

## Autoantigen arrays for profiling autoantibodies associated with complications in SLE

### Autoantibodies associated with LN

LN is a leading cause of mortality in SLE and autoantibodies constitute important contributors to renal damage in this disease. In order to better understand the seroprofile of nephrophilic autoantibodies in SLE, Li et al. constructed a multiplexed proteome microarray bearing 35 antigens known to be expressed in the glomerular milieu to investigate serum autoantibodies in both human and LN mouse model [22]. They found that LN mice (B6.Sle1.lpr) exhibited high levels of IgG and IgM antiglomerular antibodies as well as anti-dsDNA/chromatin antibodies and variable levels of antibodies to α-actinin, aggrecan, collagen, entactin, fibrinogen, hemocyanin, heparin sulfate, laminin, myosin, proteoglycans, and histones. The use of these glomerular proteome arrays also revealed five distinct clusters of IgG autoreactivity and two clusters of IgM autoreactivity in the sera of lupus patients [22]. The two IgG autoantibody clusters, DNA/chromatin/glomeruli and laminin/myosin/matrigel/vimentin/heparan sulfate, showed strong association with disease activity, whereas the IgM autoantibodies was associated with reduced disease activity [22]. Further investigation of autoantibody profiling on polycongenic mice with severe LN (B6.*Sle1Sle3* and B6.*Sle1Sle5*) revealed that B6.*Sle1Sle3* and B6.*Sle1Sle5* mice had more IgG autoantibodies of glomerular specificities than B6.*Sle1* mice, and B6.*Sle1Sle3* mice also had higher levels of IgA autoantibodies targeting dsDNA and histone, compared to B6 mice [27]. These studies suggested that the glomerular proteome array promises to be a powerful analytical tool for uncovering novel autoantibody disease associations and for distinguishing patients at high risk for end-organ disease.

Using an antigen array bearing 694 antigen specificities, Fattal et al. investigated autoantibodies in SLE patients on various clinical stages – SLE with acute LN, those in renal remission, and those who had never had renal involvement [28]. They found that SLE patients had significantly increased IgG autoantibodies against dsDNA, ssDNA, Epstein–Barr virus (EBV), and hyaluronic acid, compared to healthy controls. Moreover, the levels of these autoantibodies are persistently higher in SLE patients even after long-term clinical remission and independent of disease activity [28]. They also found that IgM reactivities to myeloperoxidase (MPO), CD99, collagen III, insulin-like growth factor binding protein 1 (IGFBP1), and cardiolipin were decreased in SLE, suggesting that the IgM autoantibodies might enhance resistance to SLE, consistent with the findings by Li and colleagues [22].

### Autoantibodies associated with neuropsychiatric SLE

Neuropsychiatric SLE (NPSLE) is an important subtype of SLE with complicated clinical manifestations, including aseptic meningitis, psychosis, and seizures, but the clinical diagnosis of NPSLE remains challenging due to lack of specific biomarkers [29]. Autoantibodies in the CSF of NPSLE patients might be directly associated with the disease status. Indeed, various autoantibodies targeting to neuronal tissue antigens, such as glutamate receptor ε2 subunit (GluRε2), ganglioside, glial fibrillary acidic protein, dsDNA, N-methyl-d-aspartate (NMDA) receptors, triose phosphate isomerase, SSA/Ro, ribosomal P protein, cardiolipin, and alpha internexin, have been identified from CSF of NPSLE patients [30]. Unfortunately, very few of these autoantibodies are specific to NPSLE. In order to identify more specific biomarkers associated with NPSLE, Hu et al. screened 29 CSF specimens from 12 NPSLE, 7 non-NPSLE, and 10 control (non-SLE) patients using a human proteome array with ∼17,000 unique full-length human proteins [31]. They identified 137 autoantigens associated with NPSLE, including anti-proliferating cell nuclear antigen (PCNA), anti-60S acidic ribosomal protein P0 (RPLP0), anti-RPLP1, anti-RPLP2, and anti-Ro/SS-A. The titers of anti-RPLP2 and anti-SS-A in CSF were significantly correlated with those in sera, suggesting that these autoantibodies may be potential CSF markers for NPSLE [31].

## Autoantigen arrays reveal autoantibodies in pediatric SLE

About 10%–20% of SLE patients have disease onset in childhood or adolescence and are treated as pediatric SLE (pSLE). pSLE patients often initially present with more active and severe disease manifestations than adults, including higher frequency of LN, which is the primary causes of morbidity and mortality in pSLE [32,33]. In order to reveal autoantibodies associated with proliferative LN and disease activity in pSLE, Haddon et al. used autoantigen microarrays composed of 140 antigens to compare the serum autoantibody profiles of 45 new-onset pSLE patients, including 23 biopsy-confirmed class III or IV proliferative nephritis and 18 without significant renal involvement, with the autoantibody profiles of 17 healthy controls. They found that titers of 55 autoantibodies were significantly higher in the sera of pSLE patients than the healthy controls. Anti-B cell-activating factor (BAFF) antibody, which was associated with active disease status, was on the list [34]. Furthermore, titers of 13 autoantibodies were significantly higher in pSLE patients with proliferative LN than those without. These included 5 antibodies targeting dsDNA, C1q, collagen IV, collagen X, and aggrecan, which are enriched in glomeruli. They concluded that autoantigen microarray is an ideal platform for identifying autoantibodies associated with both pSLE and specific clinical manifestations of pSLE [34].

## Autoantigen arrays distinguish antibodies in discoid LE

Discoid LE (DLE) is a chronic dermatological disorder presents in about 20% of SLE patients, which is usually associated with milder disease activity and lower prevalence of LN in comparison with SLE [35]. Previous studies showed that about 21%–63% of DLE patients are ANA positive, but the ANA titers were usually at low range (<1:160), compared to SLE [35]. In order to gain information on autoantibody specificities in subsets of DLE and SLE patients, Chong et al. compared the autoantibody profiles in SLE patients with DLE (DLE^+^SLE^+^) or without DLE (DLE^−^SLE^+^), as well as DLE subjects without SLE (DLE^+^SLE^−^), using an autoantigen array containing 98 autoantigens [36]. They found that autoantibodies targeting to several nuclear antigens (*e.g.*, dsDNA, dsRNA, histone H2A, histone H2B, SS-A, and ssDNA) showed distinctively lower levels in patients with DLE (DLE^+^SLE^+^ and DLE^+^SLE^−^) than in SLE patients without DLE (DLE^−^SLE^+^), implying that DLE is a phenotypic marker associated with mild systemic disease. The IgG autoantibody profile in DLE^+^SLE^−^ subjects is similar to that in HCs, although a few, including three autoantibodies against nuclear antigens (Jo-1, U1-snRNP-A, and SS-A) and two against epidermal–dermal junction proteins (HSPG and α6β4 integrin), showed slightly higher levels. However, higher level of IgM autoantibodies against αβ-crystallin, lipopolysaccharide, heat-shock cognate 70 (HSC70), and desmoglein-3 were found in DLE^+^SLE^−^ subjects, compared to DLE^−^SLE^+^ and DLE^+^SLE^+^ subjects. The IgG:IgM ratios of nuclear-specific autoantibodies progressively increased from healthy to DLE^−^SLE^+^ subjects [36], implying that lower levels of IgG autoantibodies in DLE might be associated with lower disease severity, whereas higher IgM autoantibodies against selected antigens in healthy and DLE^+^ subjects may be nonpathogenic.

## Autoantigen array for detection of cytokine-specific antibodies in SLE

Autoantibodies targeting to cytokines, chemokines, and other circulating immunologic factors have been described in SLE and other autoimmune diseases [37,38], but their profiles in SLE have not been systemically studied. Recently Utz’s group reported an autoantigen array bearing 59 unique human serum factors in addition to previously-described autoantigens for parallel detection of antibodies against known autoantigens as well as novel immunological targets [39]. By assaying the autoantibodies in sera of SLE patients, they revealed elevated levels of autoantibodies targeting several serum factors in SLE, including BAFF, TGF-β1, IL-2, IL-23, TNF, and IFN-α. Specifically, they found that anti-BAFF reactivity was positively correlated with the level of INF-signature genes, which is a hallmark feature of severe SLE [39,40]. These observations imply that elevated autoantibodies targeting to serum immunological factors may be associated with more severe inflammatory status in SLE and may serve as useful biomarkers for the evaluation of disease activities [39].

## Other applications of autoantigen arrays in SLE studies

Perhaps not surprisingly, autoantigen arrays have been widely used in SLE studies. Culton et al. screened patients’ sera using autoantigen arrays bearing 67 nuclear and glomerular autoantigens to investigate the autoantibody profiles in SLE patients with high or low CD19 expressing B cells. Their data indicate that CD19(hi) SLE patients exhibit a distinct autoantibody profile characterized by high levels of antibodies to snRNPs and low levels of anti-glomerular autoantibodies [41], suggesting that B cells play a crucial role in the determination of the autoantibody specificities. On the other hand, Silverman and colleagues are interested in finding out the genetic imprint of autoantibody repertoires in SLE using autoantigen arrays. From the autoantibody profiles of 38 SLE patients including 14 sets of SLE twins, they found that autoantibodies to the phospholipid neodeterminants, malondialdehyde (MDA), and phosphorylcholine (PC) were among the most prevalent and highly-expressed autoantibodies in SLE. The sharing of IgG autoantibody fingerprints by monozygotic twins suggests that lupus IgG autoantibodies can arise in predisposed individuals in genetically-determined patterns [42]. Another important application of autoantigen arrays in human SLE studies is to determine the autoantibody alterations during the transition from non-active to active disease status. In a study of longitudinal follow-up of 22 SLE patients, Olsen and colleagues investigated the variations of IgG and IgM autoantibody specificities in these patients along with their clinical manifestation for 2.4 years. They found that IgG but not IgM autoreactivity increased to a greater extent in the progressor group than in the non-progressor group. Progressors had significant increases in IgG anti-La/SSB and liver cytosol type 1 (LC1) autoantibodies over the period of evaluation (*P *⩽ 0.0072). These findings suggest that autoantibody profiles using an expanded array of specificities can be used to predict the risk of progressive disease in patients with lupus [43].

Autoantigen arrays have also been applied in SLE studies using animal models. Sekine et al. used autoantigen microarray technology to identify distinct autoantibody profiles in H-2 congenic MRL/lpr mice and indicated that genes encoded within MHC region plays critical role in the production of anti-SM and anti-snRNP autoantibodies [44]. To investigate the association of type 1 IFN with autoantibodies in SLE, Thibault et al. measured the autoantibody specificities in a pristine-induced lupus in type 1 IFN receptor 2 knockout mice (IFNAR2−/−) using autoantigen arrays and found that the absence of type 1 IFN could decrease the expression of nucleic acid-sensing Toll-like receptors and therefore, diminish the autoantibody production [45]. Given the fact that the high expression of IFN-genes is a signature of SLE in human, the study in the animal model has great value for the identification of the underlying molecular connections between the IFN genes and autoantibodies in SLE. A20 (also known as tumor necrosis factor, alpha-induced protein 3, TNFAIP3) is another important factors associated with immune regulation in autoimmune diseases. Tavares et al. used protein arrays to analyze the autoantibodies in sera from conditionally-knockout mice and showed that mice with A20 deficiency in B cells possessed more germinal center B cells, autoantibodies, and increased expression of anti-apoptotic proteins such as Bcl-x, alluding to the role of A20 in B cell survival and lupus [46]. Similar to the above studies, Satterthwaite’s group has also used autoantigen arrays for screening of autoreactive antibodies in genetically-modified mouse models (Lyn−/−, Btk−/−, Ets1−/−) for fine mapping of genetic interaction between Lyn, Ets1, and Btk in the control of antibody levels. Their data defined that there exist *in vivo* genetic interactions between Ets1, Lyn, and Btk, whereas disruption of the balance of these factors will lead to loss of immune tolerance and autoantibody production [47–49].

## Antigen arrays for discovery of novel autoantibody biomarkers in SLE

Other than detection of autoantibodies already identified in SLE, an important application of protein microarray is to identify novel autoantibodies associated with the pathogenesis of the disease. Huang et al. used protein arrays containing over 5000 recombinant human proteins to profile the autoantibodies in the sera of SLE patients and compared with HCs. Four novel antigens, *i.e.*, PBOV1, MORF4L1, CLIC2, and GSTP1, were identified to be potential targets of autoantibodies in SLE. They further validated by using ELISA that the positive rate of anti-CLIC2 in SLE was 28.18% and the level of anti-CLIC2 in SLE was positively correlated with disease activity in terms of SLE Disease Activity Index (SLEDAI) score and several other indexes (*P *< 0.05) [50]. Using Protoarray proteome arrays bearing over 9000 human full-length proteins, Kinloch et al. screened 25 monoclonal antibody strains cloned from activated B cells isolated from laser-captured renal biopsy specimens of LN patients. They identified that vimentin is a dominant autoantigen targeted *in situ* in LN with severe tubulointerstitial inflammation (TII) [51].

## Technological improvement of autoantigen arrays

Technologic modification has been applied to the planar array for the purpose of increasing its sensitivity on detecting novel targets. Kattah et al. described a two-color antibody-binding fragment (Fab) labeling method for protein array [52]. First, they spiked mouse antibodies into normal mouse serum, and then they pre-incubated the spiked samples with Cy3- or Cy5-labeled anti-mouse monovalent Fabs, respectively. After removing free Fabs by passing over a spin column for purification, they mixed the two samples and applied them to a protein array. Using this method, they discovered a previously-unreported reactivity to Ribo P0 in autoimmune mice. Another important modification on protein array is the improvement of coating chemistries on the array. El Khoury et al. developed newer surface chemistries for protein arrays so that the protein structure and biological activity can be retained [53]. They performed these modified arrays to evaluate the anti-histone autoantibodies present in SLE patients’ sera, and compared the results with those detected using ELISA and Western blotting. Their data indicated that arrays had a higher sensitivity than ELISA and Western blotting, and required smaller volume of samples.

## Autoantigen array data analysis

No doubt autoantigen arrays as a highly-multiplex and high-throughput assay provided much more information than traditional assays. Nonetheless, the data interpretation and cross-study comparison is a challenging topic facing bioinformatists. Unlike DNA microarrays, for which various analytical algorithms have been established for data mining and classification, so far there is not an applicable data mining pipeline available for autoantigen array data analysis. One of the inherent disadvantages of antigen microarrays is the existence of batch and inter-array variations, making it difficult to compare results obtained from different experiments and at different locations. Quantile and global normalizations, which are used to reduce inter-array variability in DNA microarray, are not readily applicable to protein array data analysis, simply because these algorithms could introduce artifacts that distort the signals and affect the identification of real protein signals [54]. To overcome this limitation, Sboner et al. reported a robust linear model (RLM) to reduce technical variability in functional protein microarrays. They incorporated a set of control proteins into subarrays as normalization factor and showed that RLM normalization was able to reduce both intra- and inter-array technical variability while maintaining biological differences [54]. Incorporation of different internal standards, such as tagged-internal standard (TIS), could facilitate the normalization and thus improve the data quality [55]. Our group has incorporated purified human Ig (IgG and IgM) and anti-human Ig (anti-human IgG and anti-human IgM) as internal controls (https://microarray.swmed.edu/products/category/protein-array/). We found that the replicated Ig protein spots incorporated into arrays generated consistent signals and are suitable for normalization between arrays. Furthermore, the signal of anti-human IgG (or IgM) antibody incorporated into each array could be used to measure the total amount of IgG (or IgM) in the sera (Li et al. unpublished).

## Conclusion

Autoantigen microarrays provide a unique tool to profile autoantibodies in systemic autoimmune diseases. SLE is an extremely-heterogeneous autoimmune disorder. The immune system creates large number of different antibodies targeting different antigens in different patients, which are in turn associated with different clinical manifestation. Thus, autoantigen array as a highly-multiplex autoantibody detection platform is extremely valuable not only in SLE studies, but also for the potentials of clinical applications. Since autoantibodies appear in the blood prior to clinical onset of the disease, detection of pathogenic autoantibodies may help to identify individuals at risk of developing SLE. Furthermore, detection of certain patterns of autoantibodies may help to monitor disease activity and to evaluate response to therapy. As more novel autoantigens/autoantibodies are being discovered and their correlation with disease development being elucidated, the use of this high-throughput technology in clinical diagnosis will be highly demanded. Nevertheless, there are still a lot of technical challenges for this technology. Firstly, the panel of antigens on the arrays and the protocol for array processing should be further standardized so that the results from different studies could be comparable. Secondly, since the variations between different batch of slides or even inter-array variability are still a big concern for the application of autoantigen arrays, an optimized data normalization algorithm is in great need. To overcome these technical limitations, the laboratory scientists are working closely with rheumatologists and bioinformatists to improve the array design and data processing algorithms. With the rapid application of multi-omics technologies on the biomarker analysis of diseases, we are anticipating that autoantigen microarrays, as a high-throughput biomarker discovery tool, should have the potential to revolutionize the diagnosis, monitoring, and therapy of SLE.

## Competing interests

The authors have declared no competing interests.

## Figures and Tables

**Table 1:** Autoantigens in SLE

**Category**	**Autoantigens**
Nuclear antigens	Double-stranded DNA, single-stranded DNA Nucleosome Chromatin Histones: total, H1, H2A, H2B, H3, H4 DNA topoisomerase I/Scl70 Centromere: centromeric protein A (CENP-A), CENP-B Proliferating cell nuclear antigen (PCNA) Ku (p70/80) Mi-2 Transcriptional intermediary factor 1 gamma (TIF1/TRIM33) Melanoma differentiation associated protein-5 (/MDA5/IFIH1) Sp100 Double-stranded RNA, single-stranded RNA Ro/Sjögren’s syndrome type A antigen (SSA): 52kDa, 60kDa La/ Sjögren’s syndrome type B antigen (SSB) Smith antigen (Sm): Sm/D, SmD1, SmD2, SmD3 Ribonucleoprotein: U1-snRNP A, B/B’, C, 68kDa Nuclear exosome: PM/Scl75, PM/Scl100 Nucleolin: C23 Ribosomal phosphoprotein: P0, P1, P2 RNA polymerase I, II, III Histidyl-tRNA synthetase/Jo-1 Threonyl-tRNA synthetase/PL-7 Alanyl-tRNA synthetase/PL-12 Signal recognition particle/SRP54

Cytoplasmic/membrane proteins	Neutrophil cytoplasmic antigens: myeloperoxidase (MPO), proteinase 3 Cytochrome P450 2D6 (LKM1) Cytochrome C Liver cytosol antigen type 1 (LC1) M2: target of antimitochondrial antibodies Tissue transglutaminase (TTG) ß_2_ microglobulin Mitochondrial antigen

Nuclear membrane-associate antigens	Nuclear pore glycoprotein 210 Nucleoporin 62kDa

Cell matrix proteins	Collagen I, II, III, IV, V, VI Fibrinogen, fibronectin

Phospholipid proteins	ß2-glycoprotein 1/ apolipoprotein H Cardiolipin Glycoprotein 2

Glomeruli-specific proteins	Glomerular basement membrane Actinin, laminin Matrigel, amyloid, elastin

Thyroid-specific proteins	Thyroid peroxidase Thyroglobulin

Circulating proteins	Complement C1q, C3, C4 Prothrombin Intrinsic factors
